# Synergy Testing of FDA-Approved Drugs Identifies Potent Drug Combinations against *Trypanosoma cruzi*

**DOI:** 10.1371/journal.pntd.0002977

**Published:** 2014-07-17

**Authors:** Joseph D. Planer, Matthew A. Hulverson, Jennifer A. Arif, Ranae M. Ranade, Robert Don, Frederick S. Buckner

**Affiliations:** 1 Department of Medicine, University of Washington, Seattle, Washington, United States of America; 2 Drugs for Neglected Diseases Initiative, Geneva, Switzerland; Northeastern University, United States of America

## Abstract

An estimated 8 million persons, mainly in Latin America, are infected with *Trypanosoma cruzi*, the etiologic agent of Chagas disease. Existing antiparasitic drugs for Chagas disease have significant toxicities and suboptimal effectiveness, hence new therapeutic strategies need to be devised to address this neglected tropical disease. Due to the high research and development costs of bringing new chemical entities to the clinic, we and others have investigated the strategy of repurposing existing drugs for Chagas disease. Screens of FDA-approved drugs (described in this paper) have revealed a variety of chemical classes that have growth inhibitory activity against mammalian stage *Trypanosoma cruzi* parasites. Aside from azole antifungal drugs that have low or sub-nanomolar activity, most of the active compounds revealed in these screens have effective concentrations causing 50% inhibition (EC_50_'s) in the low micromolar or high nanomolar range. For example, we have identified an antihistamine (clemastine, EC_50_ of 0.4 µM), a selective serotonin reuptake inhibitor (fluoxetine, EC_50_ of 4.4 µM), and an antifolate drug (pyrimethamine, EC_50_ of 3.8 µM) and others. When tested alone in the murine model of *Trypanosoma cruzi* infection, most compounds had insufficient efficacy to lower parasitemia thus we investigated using combinations of compounds for additive or synergistic activity. Twenty-four active compounds were screened *in vitro* in all possible combinations. Follow up isobologram studies showed at least 8 drug pairs to have synergistic activity on *T. cruzi* growth. The combination of the calcium channel blocker, amlodipine, plus the antifungal drug, posaconazole, was found to be more effective at lowering parasitemia in mice than either drug alone, as was the combination of clemastine and posaconazole. Using combinations of FDA-approved drugs is a promising strategy for developing new treatments for Chagas disease.

## Author Summary

Chronic infection with *Trypanosoma cruzi* causes progressive damage to the heart and other organs that is fatal in about 30% of cases. Known as Chagas disease, this is a major public health problem in Latin America. The existing medicines were developed over forty years ago and are not widely used because of toxicity and unreliable effectiveness. To discover better treatments, we screened a collection of existing drugs for growth inhibitory activity on *Trypanosoma cruzi*. Several dozen orally administered drugs were discovered, but when used by themselves they were not strong enough to cure the infection in an animal model. We tested a set of 24 of these drugs in every two-way combination and identified eight synergistic partners. At least two of these combinations were able to substantially lower parasite levels in the mouse model of *Trypanosoma cruzi* infection. Thus, finding pairs of FDA-approved drugs that can be used in combination may be a pragmatic and effective strategy for designing new therapies for Chagas disease.

## Introduction

The need for new more effective drugs to treat Chagas disease has not been matched by drug discovery efforts. An estimated 8 million people have chronic infection with the etiologic agent, *Trypanosoma cruzi* [1]. Existing treatments consist of two nitroaromatic compounds (benznidazole and nifurtimox) that are poorly tolerated and have uncertain efficacy for curing chronic infection [2]. Historically, the pharmaceutical industry has not invested substantially in tropical diseases such as Chagas disease for economic reasons. The rising costs of bringing new drugs to the market exacerbates the situation, despite the recognized expansion of Chagas disease into wealthier parts of the world [3]. No new clinical drugs for Chagas disease have been licensed or evaluated in Phase III clinical trials since the introduction of benznidazole and nifurtimox in the 1960–70's. The barriers to bringing entirely new clinical entities through preclinical and clinical development are formidable, hence, alternative strategies for Chagas disease drug development need to be considered. Repurposing existing drugs is an attractive option for “neglected tropical diseases” because the costs associated with preclinical testing and attrition are avoided and, generally, the safety profiles and pharmacological characteristics are well characterized and can be matched to the particular clinical need. Thus, it may be possible to discover licensed drugs that could be rapidly advanced to clinical trials for neglected diseases such as Chagas disease. To address this question, we combined *in vitro* screening of compounds for anti-*T. cruzi* activity with follow-up *in vivo* studies in a murine model of acute *T. cruzi* infection. This strategy has been employed by us and others leading to the discovery of various categories of drugs with anti-*T. cruzi* activity [4]–[6]. For example, antifungal agents (i.e., ergosterol biosynthesis inhibitors), tricyclic antidepressants, and various antipsychotic agents have been discovered in such screens [5]. The drug discovery efforts have led to a phase II clinical trial of the antifungal agent, posaconazole, in Chagas patients in Spain (ClinicalTrials.gov Identifier: NCT01162967), and Argentina (ClinicalTrials.gov Identifier: NCT01377480) with results yet to be published. Although azole antifungal drugs represent a potentially attractive therapeutic alternative to the existing treatment options, their efficacy for treating Chagas disease is not yet established. It is important to continue to try to identify existing drugs in hopes of repurposing them for Chagas disease.

**Figure 1:**
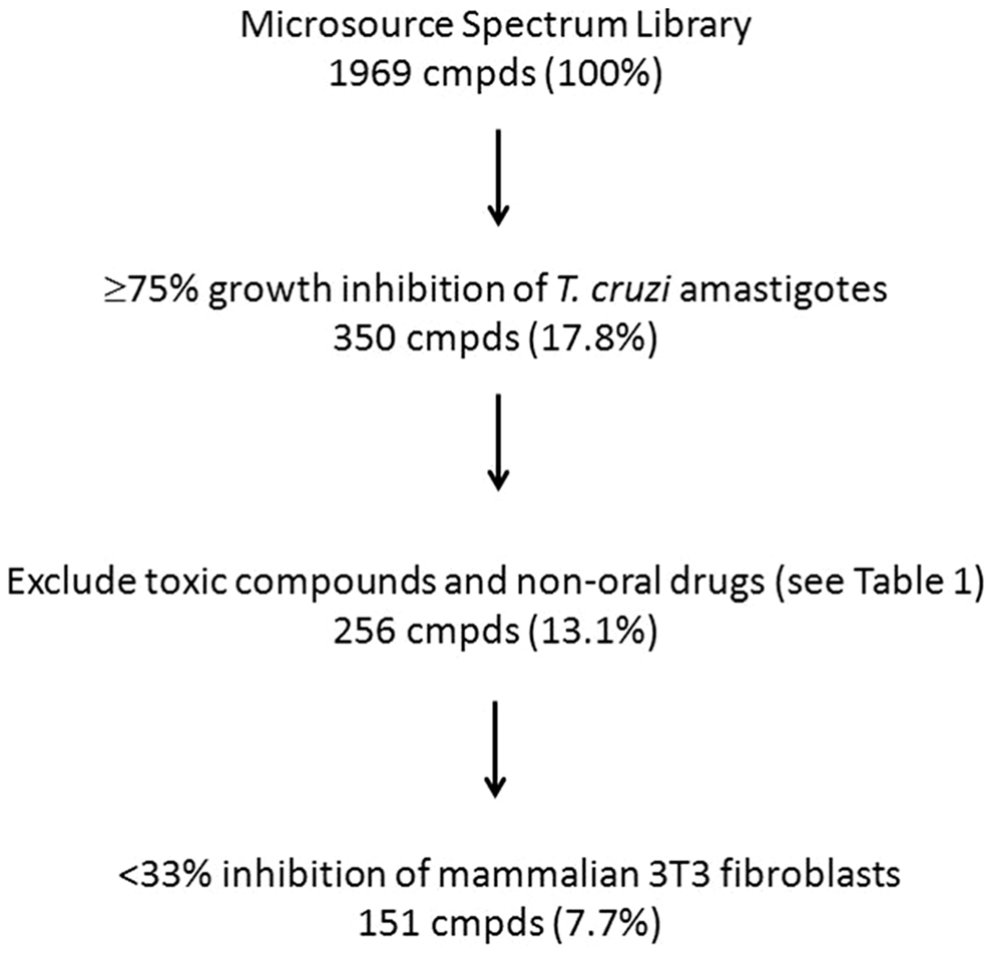
Flowchart of compound screen.

However, with the exception of azoles (and allopurinol) [7], none of the clinical drugs discovered to date has shown enough activity to lead to testing in formal clinical trials. Thus, a different strategy may be necessary to find “off the shelf” drugs that could be used for Chagas disease. In this study, we screened a collection of Food and Drug Administration (FDA)-approved drugs and biologically active compounds, and then systematically evaluated the hits from our screens in combinations searching for synergistic partners (Figure 1). A number of novel drug combinations showed *in vitro* synergy and improved survival in the mouse model of acute *T. cruzi* infection, supporting the utility of this strategy for drug development. Additional work will be necessary to establish which drug combinations may be curative in animal models and candidates for possible clinical studies.

## Materials and Methods

### Test compounds

The Spectrum Collection of 2000 biologically active, diverse compounds was purchased from MicroSource Discovery Systems, Inc. (Gaylordsville, CT) [8]. The collection includes ∼700 FDA-approved drugs. The compounds were provided as 10 mM DMSO stocks in 96-well plate format. Compounds used in synergy assays and mouse efficacy studies were purchased from Sigma-Aldrich, except as follows. JK-11 corresponds to compound **1** in a previous publication [9] and, benznidazole was acquired as previously described [10].

### Screening and EC_50_ assays

Compounds were tested against *T. cruzi* (Tulahuen strain) stably expressing the beta-galactosidase gene as previously described [11]. All *in vitro* assays were performed on mammalian-stage *T. cruzi* grown in co-culture with murine 3T3 fibroblasts using RPMI-1640 media (w/o phenol red, w/o L-glutamine) supplemented with 10% heat inactivated fetal calf serum, 100 u/mL penicillin/100 ug/mL streptomycin, 2 mM L-glutamine (final concentrations) [10]. Fibroblasts were plated at a density of 2×10^3^ per well in 96 well tissue culture plates. After 24 hours of incubation, 1×10^4^ trypomastigotes/well were added to the fibroblasts and incubated for 4 hours before addition of the test compounds from the Spectrum Collection (10 µM final concentration). Cultures were incubated at 37°C for 5 days, then developed with chlorophenol red β-D-galactopyranoside as previously described [11]. The percent inhibition is reported with standard deviation of the mean. For the effective concentration causing 50% growth inhibition (EC_50_) measurements, the compounds were tested in triplicate in serial two-fold dilutions and EC_50_ (or EC_25_) values were calculated by non-linear regression using Graphpad Prism (San Diego, CA). Similarly, for measuring the cytotoxicity concentration (CC_50_) for 3T3 fibroblast cells, cultures were incubated with drugs for 72 hours and developed using Alamar Blue (Alamar Biosciences Inc, Sacramento, CA) as previously described [11]. Z-prime values were calculated for each 96-well plate based on positive (4 wells) and negative controls (4 wells) [12].

### Combination screens

Twenty-four compounds were selected for testing in combinations. All two-way combinations were tested (300 experiments). First, EC_25_ concentrations were determined for the individual compounds against *T. cruzi* amastigotes as described above. To test for synergy, compounds were evaluated in quadruplicate individually at the experimentally determined EC_25_, and in combination with other compounds at each respective EC_25_ concentration (further explained in the Discussion section). For inclusion in downstream analysis, each individual compound in a pair was required to inhibit 25±10% of growth in positive control (untreated) wells. If not in this range, the experiment was repeated. The measured growth of *T. cruzi* amastigotes was compared to the predicted effect of the combination as follows. Assuming a simple additive effect, the predicted inhibition of the drug pairs was expected to be the product of the percent-growths of each compound when tested alone. For example, if compound A gave 75% growth of the control and compound B gave 80% of the control growth, then the combination would be predicted to be 60% (i.e., 75%×80% = 60%). With this “prediction”, we then evaluated each compound combination for whether it resulted in more or less growth than would be expected by the additive effects, and calculated a proportional effect based on the following equation.
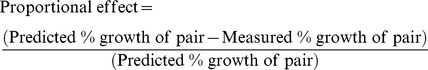



The results were tabulated and displayed in a heat-map format to help visualize the variance away from the predicted effects of the pairs. Cells in green indicate a greater effect than predicted (“synergism”) and the squares in red indicate a lesser effect than predicated (“antagonism”). A few empty boxes remain for experiments that did not meet the quality standard mentioned above despite at least two efforts.

### Isobologram studies

Drug combinations observed to have possible synergism in the screen described above were subjected to formal isobologram analysis using the fixed ratio method [13]. Drug combinations were set up with the highest concentrations in the following proportions of their EC_50_: 4∶0, 2.67∶1.33, 2∶2, 1.33∶2.67, 0∶4. Serial two-fold dilutions were performed in triplicate. Amastigote cell growth was quantified by colorimetric readout after 5 days of culture. For each ratio, an EC_50_ was calculated for each of the drugs. The fractional inhibitory concentrations (FIC) were calculated as the [EC_50_ when in combination]/[EC_50_ of drug alone]. The sum of the FIC was calculated as follows: ΣFICs = FIC drug A + FIC drug B. The mean sum of the FIC (*x*ΣFIC) was calculated as the average of ΣFIC from the three different fixed ratios. The interactions were considered synergistic for *x*ΣFIC≤0.5, indifferent for *x*ΣFIC between 0.5 and 4, and antagonistic for *x*ΣFIC>4.

### Animal efficacy experiments

**Figure 2:**
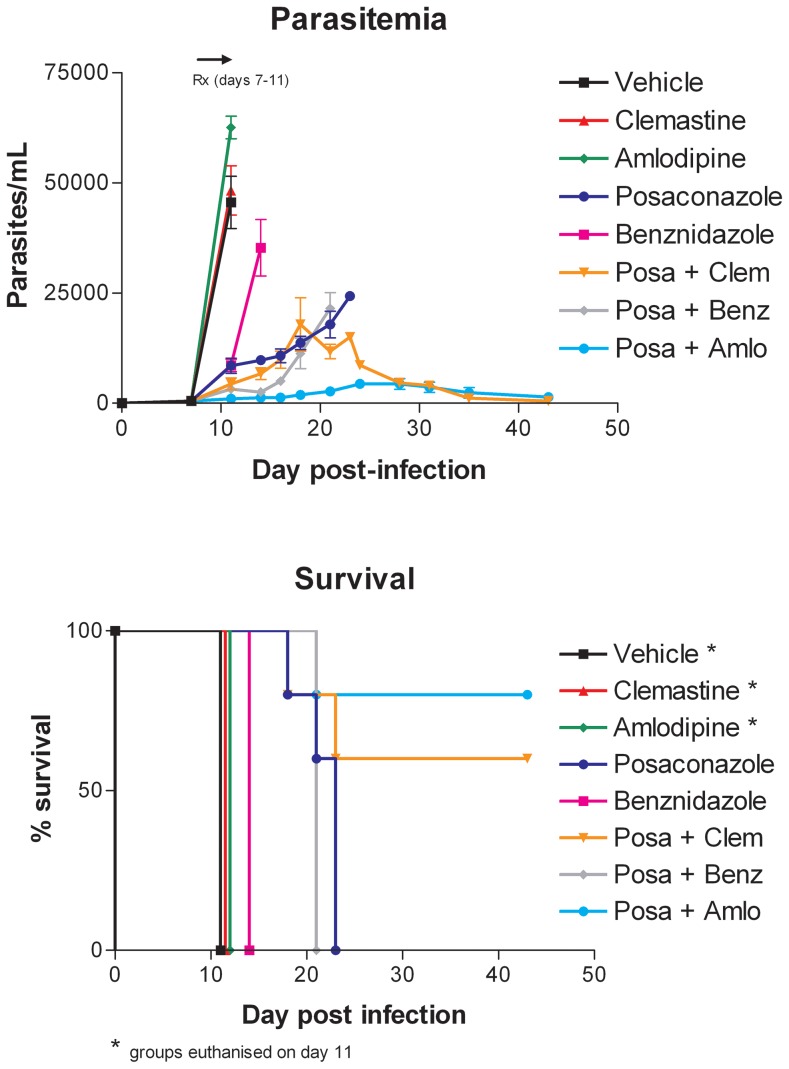
Murine efficacy study #1. Mice were infected with *T. cruzi* (1×10^4^) on day 0 and treated with the drugs (n = 6 per group) from day 7 to 11. Doses of drugs are shown in Table 5. Bloodstream trypomastigotes were quantified at the indicated time points. Mortality is plotted in the lower panels. Mice were euthanized when they showed high parasitemia and weights dropped below 20% of baseline.

**Figure 3:**
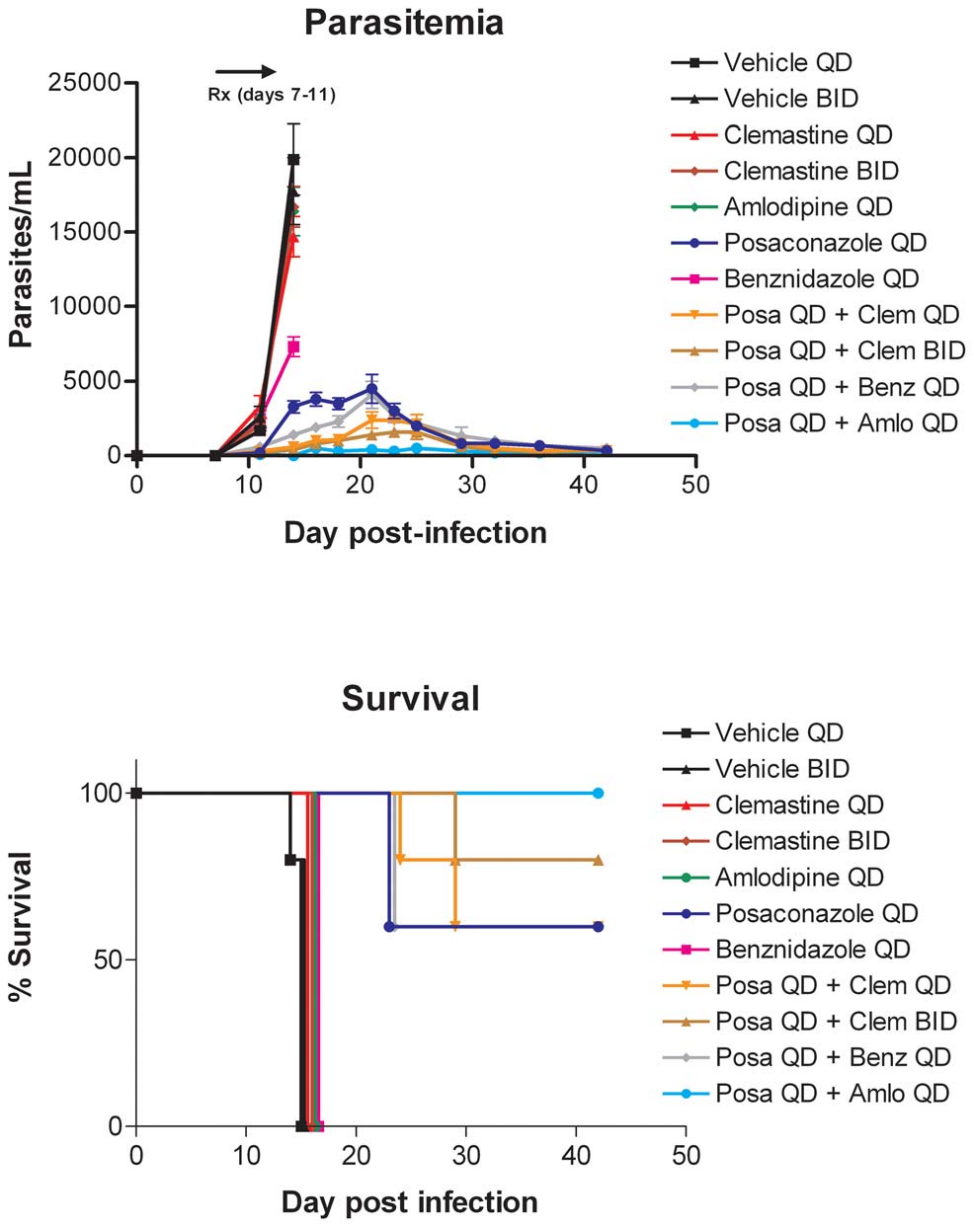
Murine efficacy study #2. As in figure 2.

Age 8–10 week-old BALB/c female mice were obtained from Harlan Laboratories. Mice were infected with 1×10^4^ tissue culture derived wild-type trypomastigotes of the Tulahuen strain by subcutaneous injection on day 0. They were administered test drugs in groups of five by oral gavage on days 7–11. All drugs were dissolved in vehicle composed of sodium carboxymethylcellulose 0.5% w/v, benzyl alcohol 0.5% v/v, Tween 80 0.4% v/v diluted in 0.9% aqueous NaCl solution. Parasitemia was quantified by examining tail blood specimens at times points indicated in Figures 2, 3, S4, and S5 as previously described [14].

### Ethics statement

All mouse work for this project was reviewed and approved by the University of Washington Institutional Animal Care and Use Committee under protocol 2154-01. The University of Washington has an approved Animal Welfare Assurance (#A3464-01) on file with the NIH Office of Laboratory Animal Welfare (OLAW), following guidelines of the USDA Animal Welfare Act and Regulations.

## Results

### Library screen against *T. cruzi*

The Spectrum Collection of 2000 compounds was screened at 10 µM against intracellular *T. cruzi* amastigotes in duplicate plates. Thirty-one compounds were not included in the screen due to precipitation. The complete ranked data set is provided in Table S1. The quality of the data was excellent as demonstrated by the Z-prime values averaging 0.65 (Figure S1). Growth inhibition of 3 standard deviations above the no-drug control corresponds with 32.1% inhibition, yielding a hit rate of 40.1% by this criterion (i.e. 791 hits, Table S1). By defining “hits” as compounds causing ≥75% growth inhibition, a subset of 350 compounds (17.8%) was identified, including all compounds above the yellow line in Table S1.

Our goal was to establish a set of compounds for characterization as potential anti-trypanosomal agents. With this in mind, we eliminated compounds that are known to be toxic or lack potential to be developed as drugs (criteria for exclusion are shown in Table 1). By applying these criteria, 94 compounds were readily removed leaving 256 (13.1%) compounds (see Table S1, column labeled “Discarded”). Examples of excluded compounds include phenylmercuric acetate (toxic) and emetine (induces vomiting). The 256 remaining compounds were next tested for growth inhibition on mammalian 3T3 fibroblasts to exclude compounds that inhibited *T. cruzi* growth due to cytotoxicity to the host cells. The average Z′-value for these assays was 0.855. There were 105 compounds that caused >33% growth inhibition of 3T3 fibroblasts, and considered cytotoxic and excluded from further analysis. The remaining 151 compounds (Table S2) represent 7.7% of the original library and are distributed amongst a variety of chemical/drug classes (Table 2). Selected compounds from Table S2 were subjected to dose response testing against *T. cruzi* amastigotes with EC_50_ values shown in Table S2 and Table 3. We prioritized a set of compounds that exhibited potency in the *in vitro* screen and represented FDA-approved drugs with substantial clinical use (to exclude poorly-characterized candidates with the potential for toxicities).

**Table 1:** Compounds excluded from further study.

Compounds containing heavy metals (mercury, arsenic, etc.)
Compounds with primary use as topical and/or ophthalmic agents
Compounds with any of the following terms in the manufacturer's description: antiinfectant, alkylating, convulsant, emetic, antiproliferative, intercalating, insecticide, acaricide, herbicide, antifeedant, cytotoxic
Compounds known to be severely hepato- or nephrotoxic
Compounds with parenteral-only administration
Compounds known to be genotoxic or teratogenic

**Table 2:** Categories of 151 hit compounds.*

Compound Class	#
Antidepressant drugs	7
Antipsychotic drugs	8
Other psychiatric drugs	3
Antihistamines drugs	5
Adrenergic drugs	3
Calcium channel blocker drugs	3
Other cardiovascular drugs	4
Non-steroidal anti-inflammatory drug	1
Hormone modulator drugs	2
Antifungal/antiparasitic drugs	14
Antineoplastics/immune suppressant drugs	3
Natural products: alkaloids, flavonoids, steroids	90
Miscellaneous synthetic compounds	8

*See Figure 1 for selection of “hits”.

**Table 3:** *In vitro* activity of compounds selected for synergy testing.

#	Molecule name	*T. cruzi* EC_50_ (nM)	Mammalian cell CC_50_ (nM)	Selectivity index
1	Amiodarone	1700	12600; 16400	8.5
2	Amitriptyline	3560; 6600	36300; 22000	5.7
3	Amlodipine	1100	13000	11.8
4	Chlorprothixene	2350; 2600	12900	5.2
5	Clemastine	440; 370	24000	59.3
6	Clomipramine	3590; 1300	11000	4.5
7	Cloperastine	5800; 5600	21400	3.8
8	Fluoxetine	5500; 3200	15800	3.6
9	Mefloquine	6100	12100	2.0
10	Minocycline	9800	>50000	>5.1
11	Paroxetine	3300; 5600	18800	4.2
12	Primaquine	300	7900	26.3
13	Pyrimethamine	3820	28500	7.5
14	Sertraline	1500; 1900	7600	4.5
15	Simvastatin	400	4000	10
16	Thioridazine	2600	6200; 8660	2.9
17	Triamterene	1660	23000; 14800	11.4
18	Allopurinol	2800	>50,000	>17.9
19	Benznidazole	650; 600	>25000	>40
20	JK11	0.55; 0.54	12433	22605
21	Pamidronate	3000	25700	8.6
22	Pentamidine	181	>50000	>276
23	Ro 48-8071	410	7400	18.0
24	Terbinafine	17440	51000	2.9

Results of separate assays are separated by semicolons. The selectivity index is based on the average mammalian cell cytoxicity concentration (CC_50_) divided by the average *T. cruzi* effective concentration (EC_50_).

Most of the compounds had EC_50_ values in the 1–10 µM range with the exception of clemastine, primaquine, and simvastatin which had high nanomolar EC_50_s. It was our judgment that the compounds probably lacked sufficient anti-*T. cruzi* potency to be curative as monotherapies in the animal model of *T. cruzi* infection. (*In vivo* data shown below supported this assumption). As a result, we turned to the possibility that some of these compounds (and several additional drugs known to have activity on *T. cruzi*) might by synergistic with each other and this could lead to combinations for effective chemotherapy. The investigations of this hypothesis are described in the following section.

### Synergy testing

Twenty-four compounds were selected for synergy testing (Table 3). These included 17 from the Spectrum Collection screen (#1–17) and an additional 7 compounds selected from the literature (#18–24). The compounds were picked for the following reasons: 1) potency in screening assays (EC_50_<10 µM), 2) orally route of administration (except for pentamidine), 3) diversity of drug class, and 4) established history of safe clinical use (exceptions being JK-11 and Ro 48-8071 which are not registered drugs). The 24 compounds in Table 3 were subjected to testing in every possible combination. The data are shown in a matrix (Figure S2) that is heat-mapped based on the “proportional effect” of the drug pairs as described in the Methods. We obtained usable synergy data for 297 of the 300 drug pairs. Of these pairs 232 (79%) showed positive proportional effects >0% and 63 (21%) showed negative proportional effects (≤0). An example of a pair showing apparent synergism is cloperastine and clemastine (proportional effect of 88%). This was calculated as follows: cloperastine alone resulted in 79% of normal growth, clemastine alone allowed for 74% of normal growth. The predicted growth is the product of these two observations (0.79*0.74 = 0.58). However, the combination actually resulted in 7% of normal growth. Using equation 1 in the Methods, the calculated proportional effect is 88% (with a maximum possible proportional effect of 100%).

### Isobologram analysis

Twenty-three combinations that appeared to show the most synergism were next tested in formal isobologram analyses in order to quantify the interactions by this standard method. The sum of fractional inhibitory concentrations (FICs) for various combinations are listed in Table 4. Eight drug combinations were confirmed to be synergistic by having the sum of FICs less than 0.5. Four of these involved the antihistamine compound clemastine and four involved the sterol 14-demethylase inhibitor JK-11. We added another sterol 14-demethylase inhibitor, posaconazole, to these combination studies since it is now of special interest in clinical trials for treatment of Chagas disease. Like JK-11, it was also found to be synergistic with clemastine. However, fourteen of the combinations had sum of FICs above the 0.5 cut-off and thus were merely additive in the interaction rather than synergistic. The isobologram graphs are shown in Supplementary material, Figure S3.

**Table 4:** Sum of FICs.

Drug A	Drug B	Average FIC
Clemastine	JK11	0.279
Amlodipine	JK11	0.367
Paroxetine	JK11	0.379
Allopurinol	JK11	0.399
Allopurinol	Benznidazole	0.405
Clemastine	Mefloquine	0.456
Clemastine	Posaconazole	0.460
Clemastine	Amiodarone	0.487
Clemastine	Clomipramine	0.551
Minocycline	JK11	0.568
Clemastine	Amlodipine	0.577
Amlodipine	Posaconazole	0.645
Cloperastine	Mefloquine	0.674
Clemastine	Allopurinol	0.760
Paroxetine	Amlodipine	0.818
Clemastine	Cloperastine	0.825
Benznidazole	Posaconazole	0.912
Sertraline	Mefloquine	0.926
Clemastine	Minocycline	1.001
Allopurinol	Posaconazole	1.174
Clemastine	Benznidazole	1.203
Mefloquine	Amiodarone	1.210
Posaconazole	Amiodarone	1.618

### *In vivo* testing of combinations

Selected drugs identified in the above screens were tested alone or in combination in the mouse model of *T. cruzi* infection. In the first experiment, we focused on posaconazole and benznidazole because of their advanced clinical status for treating Chagas disease. Since benznidazole and posaconazole are known to have curative activity as monotherapies, we used sub-curative doses so that additive or synergistic interactions could be detected when used in combinations. The other drugs were administered at doses described in the literature for treating mice. Dosing schedules are listed in Table 5. Briefly, mice were gavaged once or twice daily with a given drug or combination on days 7–11 post-infection. We conducted a second experiment (Figure 3) examining the same drugs with the purpose of confirming and expanding upon the initial results shown in Figure 2.

**Table 5:** Doses of drugs used in mouse experiments, given once per day (except where indicated in Figures) for 5 consecutive days by oral gavage.

Drug	Experiment
Vehicle (200 µL)	1, 2, 3, 4
Clemastine 5 mg/kg	1, 3
Clemastine 100 mg/kg	2, 4
Allopurinol 15 mg/kg	3
Amlodipine 10 mg/kg	1, 2, 3
Posaconazole 0.04 mg/kg	1, 2, 3, 4
Benznidazole 5 mg/kg	1, 2, 3
Mefloquine 25 mg/kg	4
Amiodarone 50 mg/kg	4
Allpurinol 15 mg/kg + Posaconazole 0.04 mg/kg	3
Clemastine 5 mg/kg + Posaconazole 0.04 mg/kg	1, 3
Benznidazole 5 mg/kg + Posaconazole 0.04 mg/kg	1, 2, 3
Amlodipine 10 mg/kg + Posaconazole 0.04 mg/kg	1, 2, 3
Allopurinol 15 mg/kg + Benznidazole 5 mg/kg	3
Clemastine 5 mg/kg + Benznidazole 5 mg/kg	3
Clemastine 100 mg/kg + Posaconazole 0.04 mg/kg	2
Clemastine 100 mg/kg + Mefloquine 25 mg/kg	4
Clemastine 100 mg/kg + Amiodarone 50 mg/kg	4
Mefloquine 25 mg/kg + Amiodarone 50 mg/kg	4

As intended, posaconazole and benznidazole given alone at the indicated doses cause some attenuation of parasitemia compared to vehicle-treated controls. Clemastine (5 mg/kg or 100 mg/kg) and amlodipine (10 mg/kg) given as monotherapies show no differences compared to the vehicle treated mice. Of the dual therapies tested, the most potent combination was the calcium channel blocker, amlodipine, plus posaconazole, which resulted in a nearly complete suppression of parasitemia and 80–100% survival (Figures 2 and 3). The combination of posaconazole plus clemastine suppressed parasitemia to a lesser extent, whereas the combination of posaconazole and benznidazole was not substantially different from posaconazole alone (Figures 2 and 3). Administering clemastine to the mice twice per day along with posaconazole was marginally better than administering clemastine and posaconazole once per day (Figure 3).

A third experiment shown with supplementary data (Figure S4) demonstrated a similar result in which posaconazole plus amlodipine is the most synergistic combination followed by a modest effect of combining benznidazole and posaconazole. In this experiment, we observed a lower mortality rate with the *T. cruzi* infection possibly due to variation with preparing or injecting the parasites. A final mouse experiment (Figure S5) investigated additional combinations as suggested by the in vitro experiments such as mefloquine plus clemastine, mefloquine plus amiodarone, and amiodarone plus clemastine. Unfortunately, none of these combinations showed any effect above vehicle treatment.

## Discussion

The Microsource Spectrum collection of 2000 compounds yielded a high hit rate in the primary screen with approximately 40% of compounds causing growth inhibition greater than 3 standard deviations above control levels. This is not surprising considering the nature of the library (known bioactive compounds) and the fact that compounds with toxicity to mammalian cells will necessarily result in inhibition of intracellular *T. cruzi* growth. We took three steps to eliminate compounds of low interest. First, we required at least 75% inhibition of intracellular growth 10 µM which we considered sufficient potency to be biologically interesting. Next we eliminated compounds that were not candidates for drug development, such as known toxins or drugs with only parenteral routes of administration (Table 1). And third, we rescreened the active compounds against host 3T3 cells to eliminate those with >33% inhibition at 10 uM and thus causing non-specific toxicity. The result was 151 compounds (7.7% of the original set) falling into a variety of categories shown in Table 2. The largest group of compounds (90) was non-drug natural products, which were not further considered for the current purposes since they are not established drugs. These compounds may remain of potential interest for *de novo* drug development or target identification. Of the remaining 61 drugs/compounds, psychotropic drugs are prominent in the hit list (Table S2). These included several phenothiazines such as thioridazine and chlorpromazine, which have been reported in other studies of trypanosomes [5], [15]–[21]. There is evidence that phenothiazines act on *T. brucei* by inhibiting trypanothione reductase [22]. Phenothiazines have been shown to cause direct lysis of *T. cruzi* trypomastigotes [23]. Further development of phenothiazines as antichagasic agents has probably not been rigorously pursued due to concerning side effects of this drug class and the narrow therapeutic window between parasite and host cytotoxicity.

Tricyclic compounds such as nortriptyline and clomipramine also appeared as hits in our screens. As with phenothiazines, these compounds have been previously reported to inhibit growth of *T. cruzi* [24], including a study showing activity of clomipramine in the mouse model of chronic *T. cruzi* infection [25]–[27]. The tricyclic antidepressants, similar to phenothiazines in structure, have also been shown to inhibit trypanothione reductase [28]. Finally, amongst psychotropic drugs, three selective serotonin reuptake inhibitors (SSRI) had selective anti-*T. cruzi* activity: fluoxetine, paroxetine, and sertraline. The EC_50_ values were fairly modest, in the 2–6 µM range, which suggests that on their own they may not be sufficiently potent to be used as anti-*T. cruzi* agents since therapeutic blood levels of these drugs in humans are typically in the 0.1–2 µM range and they tend to be highly protein bound (information from package inserts). There is at least one other study reporting an SSRI (fluoxetine) with anti-*T. cruzi* activity (EC_50_ = 7 µM) [5].

Among antihistamine drugs some familiar compounds such as azelastine (EC_50_ = 2.2 µM) and clemastine (EC_50_ = 0.4 µM) were identified in the screens. Azelastine was also identified in the high-throughput screen by Engel et al. [5]. Such compounds are interesting because of their favorable safety profile (they are used as over-the-counter drugs) although at normal doses blood levels are probably not high enough to mediate potent anti-parasitic activity. The idea of combining antihistamines with anti-*T. cruzi* activity with drugs such as nifurtimox has appeal since it is common that antihistamines need to be provided to control side effects such as skin reactions.

Several cardiovascular drugs were also identified in the screen, including the dihydropyridine calcium channel blockers nicardipine (EC_50_ = 5.9 µM) and amlodipine (EC_50_ = 1.1 µM). These have been previously reported to show inhibitory activity against both *Leishmania* species and *T. cruzi* with a selectivity index over mammalian cells around 7–9 [29]. A mechanism of action has not been defined. Prazosin and reserpine also had EC_50_ values slightly less than 10 µM in our screen. Since therapeutic levels of these drugs in humans are lower than these EC_50_ values, it is unlikely that they could be effective when used alone for treating *T. cruzi* infection. Finally, the antiarrhythmic drug, amiodarone, was identified in the screen. This drug was previously reported to have intrinsic anti-*Trypanosoma cruzi* activity [30], [31], which is particularly fortuitous since amiodarone is frequently used to help manage the arrhythmias that are common in Chagas disease. There is evidence that amiodarone inhibits an enzyme in the ergosterol biosynthesis pathway (oxidosqualene cyclase) and has synergistic activity with posaconazole [30]. With all of these cardiovascular drugs, there needs to be special caution when considering their use in patients with Chagas disease due to the potential to exacerbate underlying cardiac problems.

Not surprisingly, several of the antifungal drugs in the library were the most potent compounds in the screen including ketonazole (EC_50_ = 0.001 µM) and amphotericin B (EC_50_ = 0.04 µM). Azole drugs such as ketoconazole bind the sterol C14-demethylase enzyme (CYP51) and inhibit sterol biosynthesis [32]. Amphotericin B is thought to act by binding to ergosterol [33], a sterol that is not present in mammalian cells but is a critical component of the *T. cruzi* cell membrane. A liposomal preparation of amphotericin B was shown to have suppressive *in vivo* activity in mice with *T. cruzi* infection [34], but further development for treating human Chagas disease has not been pursued. As discussed in the Introduction, the repurposing of azole antifungal drugs for Chagas disease, in particular posaconazole, is now in human clinical trials [35].

The following antimalarial drugs were identified in the screen: mefloquine, primaquine, artemisinin, hydroxychloroquine, and pyrimethamine. Mefloquine has been shown to have anti-*T. brucei* activity in the mouse model [36], but we are unaware of data for *T. cruzi*. The 8-aminoquinolone compound class (including primaquine) has previously been tested against trypanosomatid parasites, including *T. cruzi* [37]–[42]. Beyond studies in the mouse model of *T. cruzi* infection [40], further investigations for use in Chagas disease have not been published. Artemisinins have also been previously shown to have *in vitro* activity against *T. cruzi* and *T. brucei* in the low micromolar range [43], but further development has not been reported. Pyrimethamine (EC_50_ of 3800 nM) is a known inhibitor of dihydrofolate reductase-thymidylate synthase which has been shown to be essential in the African trypanosome [44]. Pyrimethamine was not particularly potent against *T. brucei* with an EC_50_ of 17 µM [44], but due to the lower EC_50_ on *T. cruzi* further investigation may be warranted.

Three more compounds from the screen merit further discussion: triamterene, oxyphenbutazone, and minocycline. Triamterene (EC_50_ of 1660 nM) is a widely used diuretic that blocks the epithelial sodium channel in the renal collecting tubule. It also is an inhibitor of folate metabolism [45] and has been shown to have modest activity (48 µM) against *T. brucei* but we have not found reports of it being tested against *T. cruzi*. Oxyphenbutazone (EC_50_ of 12,000 nM) is an active metabolite of the nonsteroidal anti-inflammatory drug phenylbutazone which is used for veterinary purposes but not in humans due to risk of agranulocytosis. Its activity against *T. cruzi* has not been previously reported to our knowledge. Finally, the antibiotic minocycline was found to have an EC_50_ of 9800 nM in our assay. This drug has been described to have activity in the mouse model of *T. brucei* infection [46], [47]. The related drug, tetracycline, has little or no inhibitory activity on trypanosomes (in the low micromolar concentrations used for the tetracycline inducible genetic systems for studying the trypanosomes). The mechanism of action of minocycline in *T. cruzi* is unknown, but it could potentially bind the small subunit of the kinetoplast ribosome a similar mechanism to its effects in prokaroytes [48].

From the subset of 53 active drugs (Table S2) we selected 17 for synergy testing (Table 3, #1–17). Another 7 drugs/compounds of particular interest were added to the list (Table 3, #18–24). These included the clinical drug for Chagas disease, benznidazole. Considering the well-described problems with tolerability and efficacy of benznidazole, we were interested in establishing whether a second drug could be combined with a lower dose of benznidazole to improve efficacy. We nominated several drugs that target the sterol biosynthesis pathway, which is a well validated therapeutic target in *T. cruzi* [49]. These compounds included our preclinical candidate (JK-11) that inhibits CYP51 (sterol C14α-demethylase) [9], as well as the bisphosphonate drug, pamidronate, that inhibits farnesyl pyrophosphate synthase [50], the allylamine antifungal drug, terbinafine, which inhibits squalene epoxidase [51], and the oxidosqualene cyclase inhibitor, Ro 48-8071 [10], [52]. We also included pentamidine in the list. Pentamidine's mechanism of action is not entirely clear, but it is used clinically for African trypanosomiasis and leishmaniasis, and has oral analogs under development for trypanosomiasis [53]. These 24 compounds were tested for synergy in two-way combinations under strict conditions. In this assay, compounds were tested in quadruplicate at their EC_25_ concentrations both individually (to confirm that the compound was accurately assayed at its EC_25_) and in combination. We chose to study the selected compounds at the EC_25_ for two reasons. First, this concentration results in parasite growth inhibition that is substantially greater than the intrinsic (baseline) variance of the assay. Second, the relatively low concentration at the EC_25_ allows for a large range of growth inhibition to be observed such that synergistic activity can be detected if it is present.

More than 75% of the combinations showed positive interactions (green in the heat map, Figure S2) meaning that the combined effects were more than predicted by the equation shown in the Methods section. This does not necessarily mean that the interaction reached the level of being “synergistic”. Using the relative proportional effects in Figure S2, we ranked compound combinations for synergy potential. Based on the rankings, we were able to prioritize specific drug pairings for isobologram analysis.

Twenty-four combinations were tested on *T. cruzi* using the fixed-ratio method with results shown in Table 4. Eight drug combinations had an average FIC<0.5, which is considered “synergistic”, and all but four of the 23 had average FIC values <1.0. The four combinations with the lowest average FIC values included JK-11 as one of the paired drugs. Similarly, clemastine also appeared in 4 of the combinations reaching “synergy” levels. Unfortunately, clemastine does not appear to be synergistic with benznidazole with an average FIC of 1.20. In these experiments, we also investigated posaconazole which has the same target of action (the CYP51 enzyme) as JK-11. Both posaconazole and JK-11 were synergistic with clemastine, but the combination of posaconazole and amlodipine did not reach the synergy level (average FIC 0.645) that was observed with JK-11 and amlodipine (average FIC 0.367). Surprisingly, we did not observe synergy between posaconazole and amiodarone (average FIC 1.62), which had previously been shown to be synergistic [30]. This finding may be due to the use of different parasite strains, host cells, incubation times, or other experimental variables. Finally, the combination of posaconazole and benznidazole showed an average FIC of 0.91. Although this is not “synergistic”, the interaction falls in the “additive” range and reinforces the notion of testing these two drugs together as has been reported in mouse model [54] and in a clinical trial underway in Argentina (http://clinicaltrials.gov/show/NCT01377480).

Based on these results, we decided to test various drugs alone and in combinations in the mouse model of acute *T. cruzi* infection. Aside from benznidazole and posaconazole, none of the drugs had dramatic effects on parasitemia when used alone (although there were slight effects observed with allopurinol and amlodipine, Figure S4). This supported our view that these drugs would need to be tested in combination with other drugs in order to generate significant inhibitory effects *in vivo.* The most effective combination was posaconazole plus amlodipine (Figure 2), a result we confirmed in additional experiments (Figure 3 and S4). Parasitemia was dramatically suppressed in mice treated with amlodipine plus posaconazole or clemastine plus posaconazole, but was not completely eliminated with these combinations at the doses used. Since posaconazole was dosed well below the maximum tolerated dose, parasitemia was only partially suppressed by the posaconazole part of the combination. In vitro, the combination of posaconazole plus amlodipine was borderline synergistic (average FIC 0.645), thus it is possible that a biological interaction is occurring that results in the favorable combined effect on parasitemia of these two drugs in vivo. However, it is also known that both of these drugs are metabolized by a common liver enzyme, CYP3A4, thus it is also possible that the interaction is pharmacological in that amlodipine may be boosting blood/tissue levels of posaconazole (or vice versa). The strategy of using pharmacological interactions to boost drug activities is being seen more commonly, for example with the use of ritonavir or cobicistat in antiretroviral combination therapies involving protease inhibitors [55]. Further studies will be necessary to better characterize the interaction of posaconazole and amlodipine.

The combination of posaconazole and clemastine boosted suppression of parasitemia (Figure 2 and 3). It is not clear if there is a pharmacological interaction between these two drugs in mice or *in vivo* synergy on the parasites. This combination was synergistic in vitro with an FIC of 0.46. The combination of posaconazole and benznidazole showed only a modest boost in parasitemia suppression in both experiments (Figure 2, 3, and S4), somewhat less favorable than described in another recent report [54].

Some combinations that were synergistic *in vitro* did not demonstrate similar effects *in vivo* such as clemastine + mefloquine and clemastine + amiodarone (Figure S5). It seems most likely that sufficiently high blood and tissue levels are not being achieved or maintained to produce the desired effect, but further investigation is needed. We also looked at some combinations that were merely additive *in vitro*, but nonetheless seemed like interesting partners to test *in vivo*, such as allopurinol plus posaconazole. We did not observe a positive interaction with this combination (Figure S4). Similarly, the combination of benznidazole and clemastine did not appear to show a positive interaction in mice (Figure S4) nor did the combination of mefloquine and amiodarone (Figure S5).

These experiments show positive interactions between some well-established drugs in a mouse model of acute *T. cruzi* infection. Many more combinations that were identified in the *in vitro* experiments have yet to be tested *in vivo*, so future studies may reveal even more potent drug combinations. Future studies will also need to focus on whether the combination chemotherapy can lead to parasitological cures in mice. As noted above, we deliberately used low doses of benznidazole and posaconazole in these studies in order to facilitate observing effects on bloodstream parasitemia. In future studies, we plan to determine if combining off-the-shelf drugs can allow us to use shorter courses or lower than maximum doses of benznidazole or posaconazole to cure *T. cruzi* infected mice. The ultimate goal would be to identify new treatments based on combination therapy that are more effective, better tolerated, and simpler to administer than current regimens for treating Chagas disease.

## Supporting Information

Figure S1 **Z′-prime scores of 96-well plates from screen of Microsource compound library.**
(DOCX)

Figure S2 **Synergy matrix (green indicates synergy, red indicates antagonism).**
(DOCX)

Figure S3 **Isobolograms of drug combinations on intracellular** ***T. cruzi*** **amastigotes.**
(DOCX)

Figure S4 **Murine efficacy study #3.** As in Figure 2, mice were infected with *T. cruzi* (1×10^4^) on day 0 and treated with the drugs (n = 5 per group for all experiments) from day 7 to 11. Doses of drugs are shown in Table 5. Bloodstream trypomastigotes were quantified at the indicated time points. Mice were euthanized when they showed high parasitemia and weights dropped below 20% of baseline. Note that we did not observe mortality from *T. cruzi* infection in this experiment most likely due to small variance in the infection procedures.
(TIF)

Figure S5 **Murine efficacy study #4.** As in Figure 2, mice were infected with *T. cruzi* (1×10^4^) on day 0 and treated with the drugs (n = 5 per group for all experiments) from day 7 to 11. Doses of drugs are shown in Table 5. Bloodstream trypomastigotes were quantified at the indicated time points. Mortality is plotted in the lower panels. Mice were euthanized when they showed high parasitemia and weights dropped below 20% of baseline.
(TIF)

Table S1 **Complete results from screen of 1976 compounds on** ***T. cruzi*** **and 3T3 fibroblasts.**
(XLSX)

Table S2 **151 hit compounds (see** **Figure 1** **) organized by class/mechanism.**
(XLSX)
