# Supplementary figures and images for: Synergy Testing of FDA-Approved Drugs Identifies Potent Drug Combinations against Trypanosoma cruzi
Source: PLoS Negl Trop Dis. 2014 Jul 17;8(7):e2977. doi: 10.1371/journal.pntd.0002977 (PMC4102417; doi:10.1371/journal.pntd.0002977)

**Fig. S1.** Z’-prime scores of 96-well plates from screen of Microsource compound library.


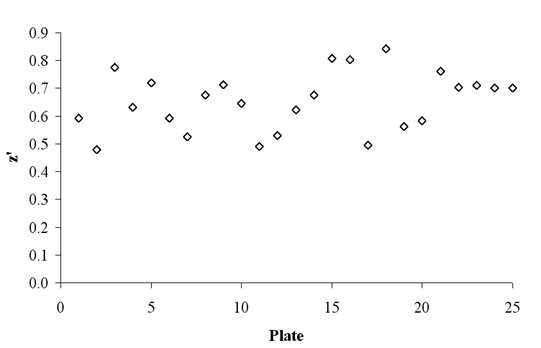

Supplement: Figure S1 — Z′-prime scores of 96-well plates from screen of Microsource compound library. (DOCX) [file pntd.0002977.s001.docx]

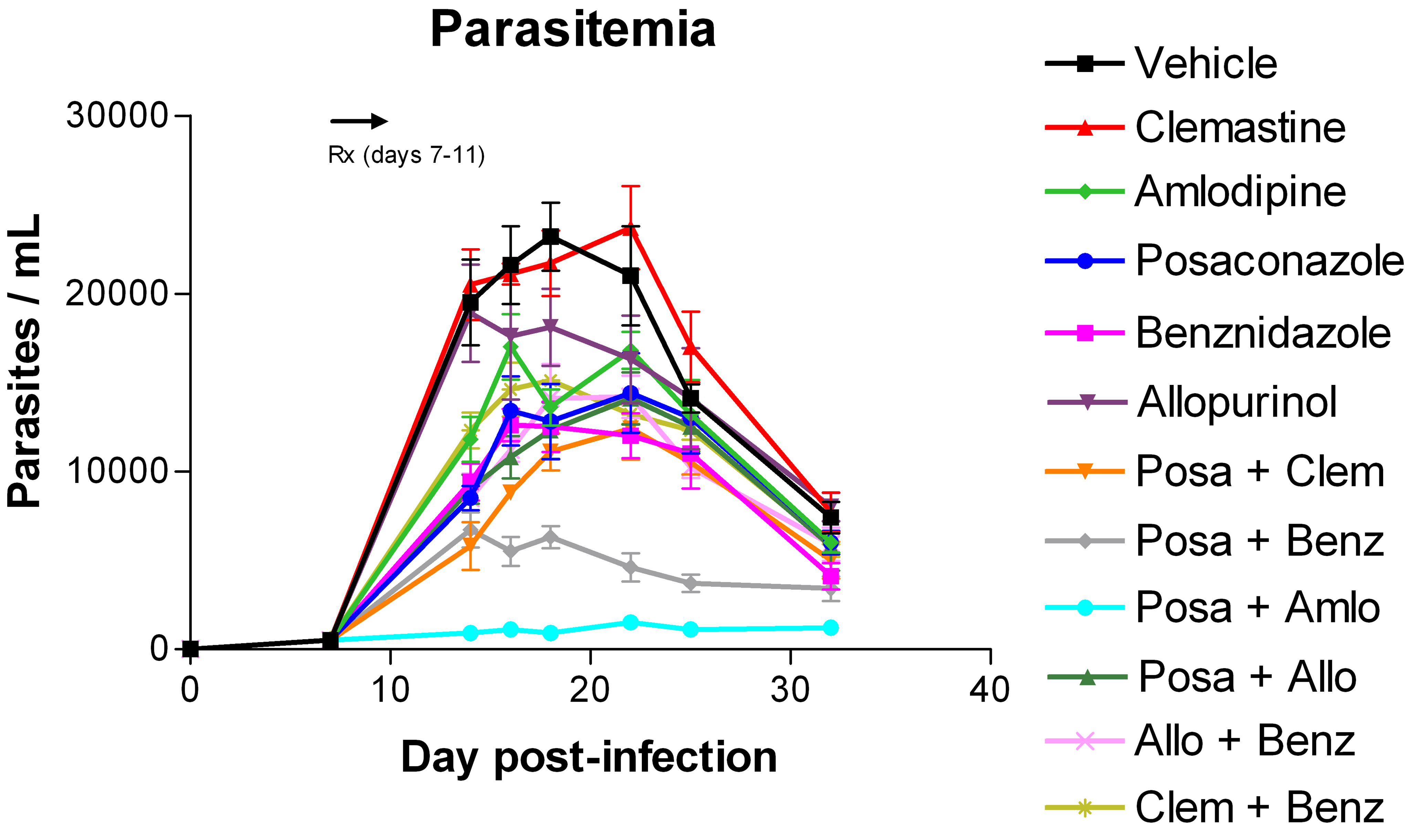

Supplement: Figure S4 — Murine efficacy study #3. As in Figure 2, mice were infected with T. cruzi (1×10^4^) on day 0 and treated with the drugs (n = 5 per group for all experiments) from day 7 to 11. Doses of drugs are shown in Table 5. Bloodstream trypomastigotes were quantified at the indicated time points. Mice were euthanized when they showed high parasitemia and weights dropped below 20% of baseline. Note that we did not observe mortality from T. cruzi infection in this experiment most likely due to small variance in the infection procedures. (TIF) [file pntd.0002977.s004.tif]

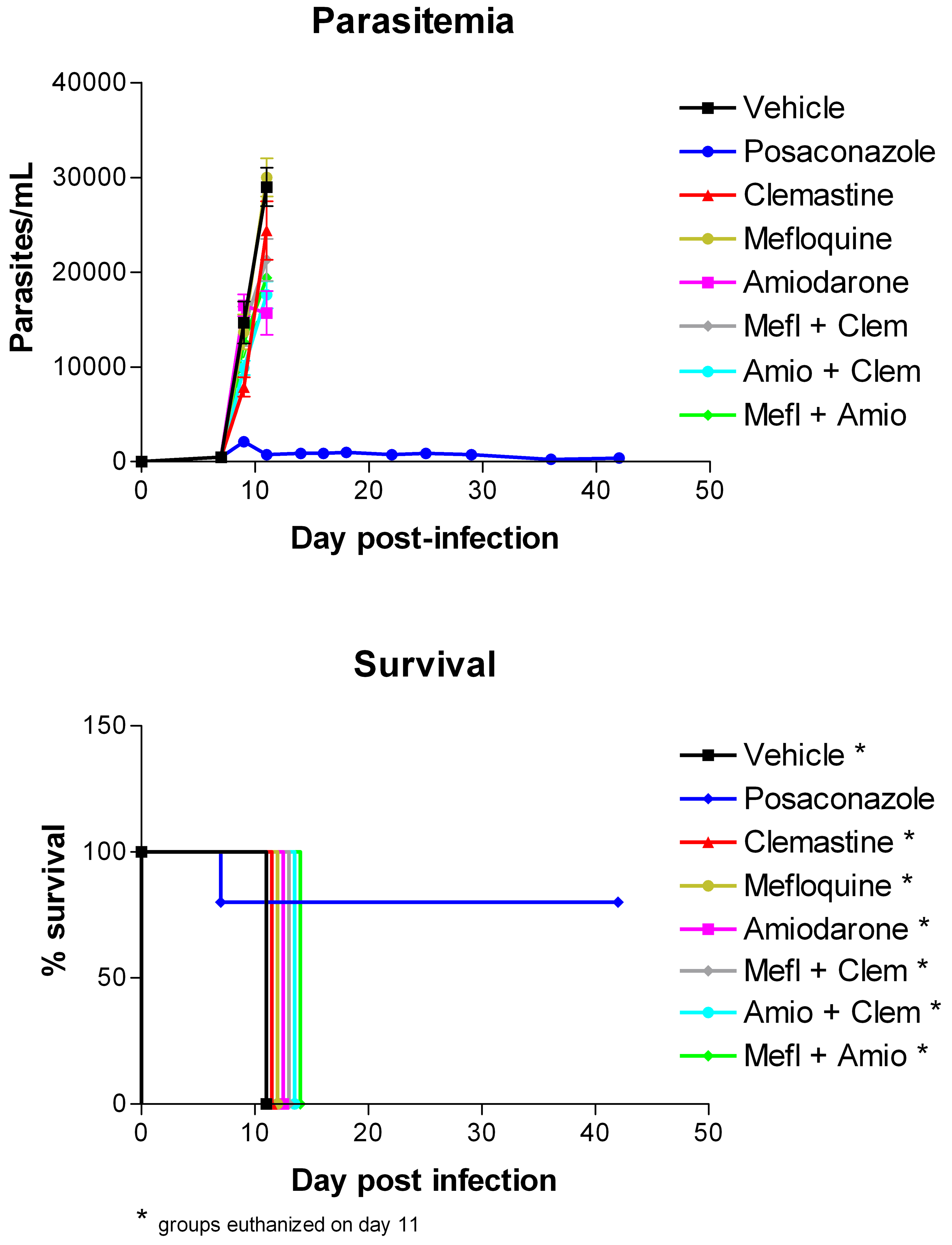

Supplement: Figure S5 — Murine efficacy study #4. As in Figure 2, mice were infected with T. cruzi (1×10^4^) on day 0 and treated with the drugs (n = 5 per group for all experiments) from day 7 to 11. Doses of drugs are shown in Table 5. Bloodstream trypomastigotes were quantified at the indicated time points. Mortality is plotted in the lower panels. Mice were euthanized when they showed high parasitemia and weights dropped below 20% of baseline. (TIF) [file pntd.0002977.s005.tif]
