# Supplementary material for: Synergy Testing of FDA-Approved Drugs Identifies Potent Drug Combinations against Trypanosoma cruzi
Source: PLoS Negl Trop Dis. 2014 Jul 17;8(7):e2977. doi: 10.1371/journal.pntd.0002977 (PMC4102417; doi:10.1371/journal.pntd.0002977)
Supplement: Figure S2 — Synergy matrix (green indicates synergy, red indicates antagonism). (DOCX) [file pntd.0002977.s002.docx]

Figure S2. Synergy matrix (green indicates synergy, red indicates antagonism)
